# Sarcomatoid carcinoma of the gallbladder: A rare form of gallbladder cancer

**DOI:** 10.1177/2050313X20906739

**Published:** 2020-02-07

**Authors:** Mehdi Siddiqui, Sheetal Hegde, Tung Nguyen, Scott DePaul

**Affiliations:** 1 Division of General and Hospital Medicine, Department of Medicine, University of Texas Health Science Center at San Antonio, San Antonio, TX, USA; 2 Department of Medicine, University of Texas Health Science Center at San Antonio, San Antonio, TX, USA; 3 South Texas Veterans Health Care System, San Antonio, TX, USA

**Keywords:** Sarcomatoid, gallbladder, carcinosarcoma, adenocarcinoma

## Abstract

Sarcomatoid carcinoma of the gallbladder or gallbladder carcinosarcoma is an exceedingly rare malignancy. Unfortunately, patients typically present with advanced disease at diagnosis. Symptoms may include abdominal pain, jaundice, anorexia, nausea, weight loss, and a palpable abdominal mass. This malignant tumor has a poor prognosis, and treatment options include surgical resection, radiation, and chemotherapy. We detail the case of a 57-year-old male who presented with diffuse abdominal pain and jaundice. Computed tomography scan of the abdomen and pelvis showed a large mass within the gallbladder, intrahepatic ductal dilation, gastrohepatic lymph node enlargement, and liver lesions concerning for metastatic disease. A core needle biopsy from one of the liver lesions revealed poorly differentiated sarcomatoid carcinoma of the gallbladder. He was assessed to have stage IV disease and deemed not to be a surgical candidate. Palliative chemotherapy was planned; however, treatment was never started due to the development of cholangitis with sepsis. The patient ultimately opted for hospice care and passed away shortly thereafter.

## Introduction

Gallbladder carcinoma is an extremely rare malignancy with an annual incidence in the United States of 1–2 cases per 100,000 persons.^1^ The vast majority of gallbladder malignancies are adenocarcinomas, with less than 1% being sarcomatoid gallbladder carcinoma, also known as gallbladder carcinosarcoma. In fact, to date, only 108 cases of gallbladder carcinosarcoma have been reported in the medical literature worldwide.^2^ Patients typically present with abdominal pain, jaundice, anorexia, nausea, weight loss, and occasionally a palpable abdominal mass.^3^

Gallbladder carcinosarcoma is more common in females and presents at a mean age of 68.8 years. Japanese ethnicity is associated with longer survival (mean = 19.9 vs 11.5 months in non-Japanese). These tumors have a poor prognosis (median survival = 5.5 months) and often metastasize to the liver, lymph nodes, and peritoneum.^4^

## Case report

A 57-year-old homeless, Caucasian male with no known significant past medical history presented to an outside emergency department with 2 months of diffuse abdominal pain, nausea, vomiting, decreased appetite, and 40 pound weight loss. Prior to these symptoms, he was a physically active, lifetime nonsmoker with no history of alcohol or illicit drug abuse.

**Figure 1.**
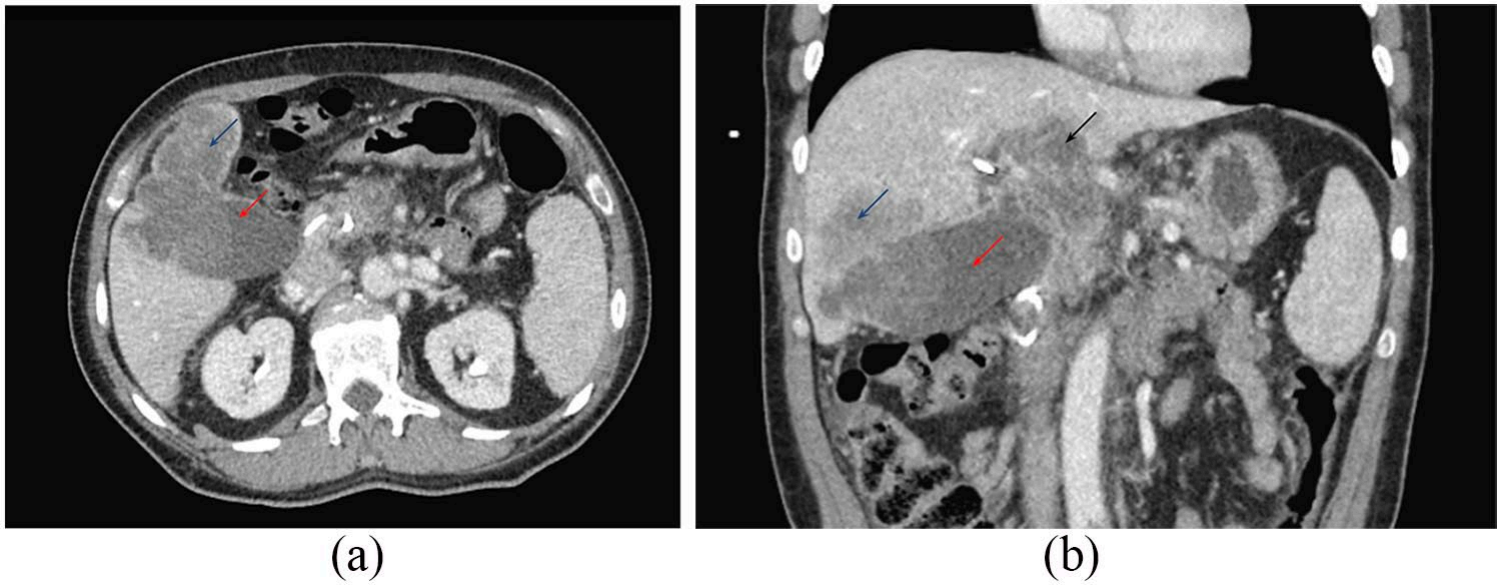
(a) CT scan at date of admission. Gallbladder lesion within the fundus measures up to 6.2 cm (red arrow). Hepatic lesion (blue arrow) adjacent to the gallbladder mass probably related to local invasion. (b) Coronal CT view shows gallbladder lesion within the fundus measures up to 6.2 cm (red arrow) and hepatic lesion (blue arrow) are again demonstrated. Further adjacent metastasis in the left hepatic lobe (black arrow) is seen.

**Figure 2.**
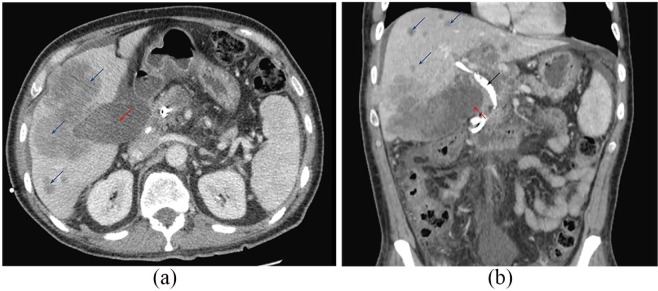
(a) CT scan at 3 week after admission. Gallbladder lesion (red arrow). There is increased size and number of hypodense lesions (blue arrows) suggestive of progressive disease. (b) Coronal CT view. Primary gallbladder lesion (red arrow). Multiple new small hypodense metastasis lesions (blue arrows) scattered throughout the liver. Increased size of previously seen local invasions. Percutaneous transhepatic biliary drain (black arrow) is seen.

**Figure 3.**
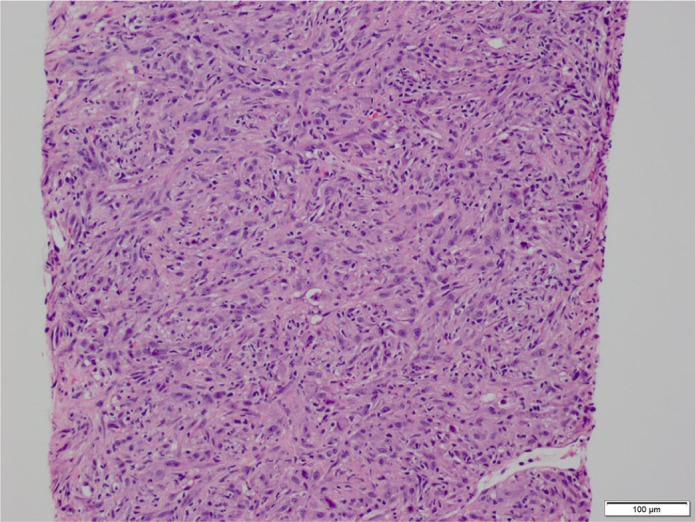
This is hematoxylin and eosin stain showing disrupted architecture, pleomorphic nuclei, and mitotic activity. These findings are of the entire slide (no part of the slide is normal).

Physical exam revealed diffuse jaundice and abdominal tenderness. His labs were significant for an elevated alkaline phosphatase of 195 IU/L and total bilirubin of 10.0 mg/dL. Computed tomography (CT) scan of the abdomen and pelvis showed a 6.2-cm mass within the gallbladder with intrahepatic ductal dilation, an adjacent 4.7-cm mass with gastrohepatic lymph node enlargement, and numerous liver lesions concerning for metastatic disease (Figure 1(a) and (b)). Due to the concern for malignancy, a biopsy of one of the liver lesions was performed. Pathology of the liver lesion revealed poorly differentiated sarcomatoid carcinoma of the gallbladder (Figures 2 and 3). The patient was diagnosed with stage IV disease, deemed not to be a surgical candidate. Endoscopic retrograde cholangiopancreatography (ERCP) was attempted in an effort to decompress the biliary system, but was unsuccessful. A percutaneous transhepatic cholangiogram was then conducted with placement of an internal-external biliary drainage catheter, after which the total bilirubin dropped to 2.4 mg/dL and the patient had symptomatic improvement.

The patient was subsequently transferred to our hospital where he arrived with worsening right upper quadrant (RUQ) abdominal pain, jaundice, and nausea. Physical exam revealed hepatomegaly, RUQ firmness, and generalized abdominal tenderness on palpation. CT of the abdomen and pelvis redemonstrated the large gallbladder fundus lesion with porta hepatis and periportal lymphadenopathy, local tumor invasion, liver metastases, as well as gastrohepatic and retroperitoneal lymphadenopathy.

**Figure 4.**
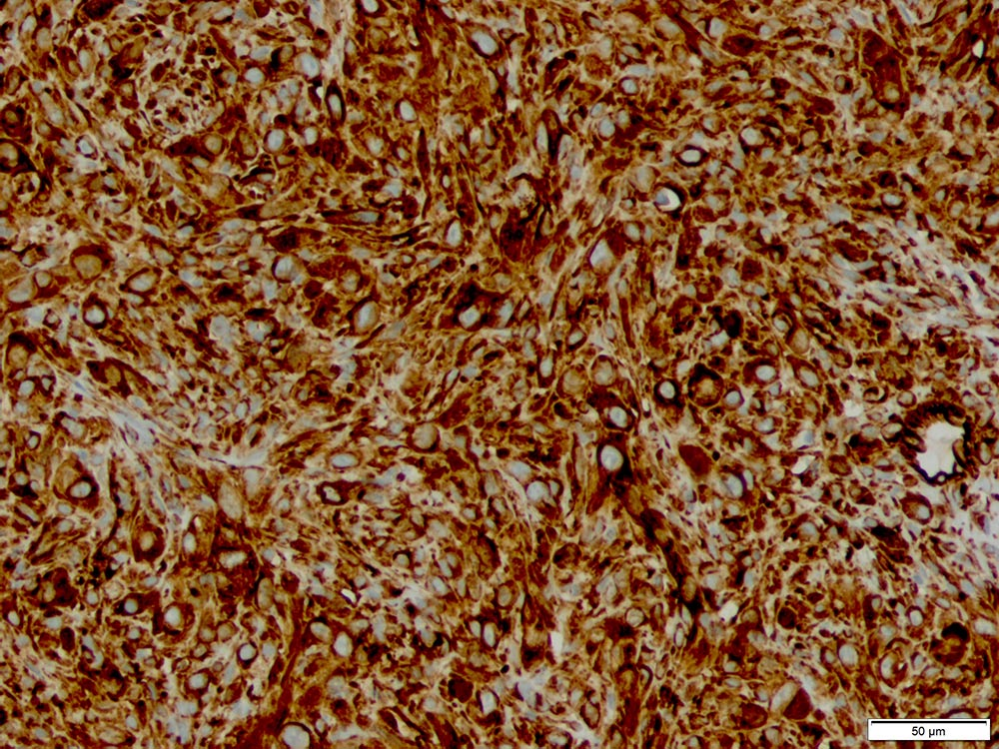
This is an immunohistochemistry stain which is diffusely and strongly positive for vimentin.

Oncology was consulted and initially recommended palliative chemotherapy with doxorubicin (adriamycin), ifosfamide, and mesna (AIM regimen). The day after port placement for chemotherapy, though, the patient developed fevers with worsening abdominal pain and was started on broad-spectrum antibiotics given the concern for sepsis due to cholangitis. Culture of biliary fluid indicated heavy growth of Candida albicans, Enterococcus faecalis, Enterobacter cloacae, Klebsiella oxytoca, and Enterococcus casseliflavus. Infectious disease was consulted and his antibiotic regimen was adjusted per their guidance. Due to the active infection, chemotherapy was delayed. Subsequent labwork revealed an uptrending bilirubin, prompting a repeat CT followed by a magnetic resonance cholangiopancreatography (MRCP). Sadly, these studies demonstrated progressive local tumor invasion with extrahepatic obstruction as well as an increased number of metastatic liver lesions (Figures 4A and B).

Repeat cholangiogram showed flow only to the external portion of the biliary drain; the internal portion now completely occluded by tumor. Unfortunately, his clinical status continued to deteriorate with worsening abdominal pain and rising bilirubin. Ultimately, he opted for hospice care and was transferred to inpatient hospice where he passed away shortly thereafter.

## Discussion

Gallbladder carcinosarcoma can be a diagnostic challenge due to its non-specific presentation. This malignant tumor is more common in females and presents at a mean age of 68.8 years.^4^ CT of the abdomen and pelvis is an important part of the workup as it can elucidate the size of the tumor and metastasis to nearby organs. Diagnosis of sarcomatoid carcinoma of the gallbladder is ultimately made with histopathology, which shows tumor composed of both malignant epithelial and mesenchymal (sarcomatous) components.

A meta analysis of 67 patients with sarcomatoid carcinoma of the gallbladder by Zhang et al. found the epithelial component was most commonly adenocarcinoma (79.2%) and least commonly squamous cell carcinoma (9.4%). The mesenchymal component was most commonly spindle cell type (44.6%) and least commonly osteoid (5.4%). The average tumor size was found to be 6.9 cm. Gallstones are reported in over 70% of cases of gallbladder carcinosarcoma. Though not associated with specific findings on imaging or tumor markers, it is typically much larger in size than a gallbladder adenocarcinoma.^3,4^ Prognostic factors associated with improved survival include tumor size less than 5.0 cm, early-stage disease, and Japanese ethnicity. Median survival nevertheless remains poor at only 5.5 months.^4^

The first-line treatment for gallbladder carcinosarcoma is surgical resection; however, due to the often advanced state of the disease at diagnosis, this is frequently not possible.^5,6^ Cholecystectomy is performed when tumor is confined to gallbladder, while locoregional spread of disease may necessitate resection of liver and adjacent tissues as well as lymph node dissection. Despite resection, postsurgical prognosis remains poor with high mortality due to micrometastasis and local recurrence. Okabayashi et al.^6^ examined 36 patients with carcinosarcoma who underwent surgical resection with intent to cure and found the 1, 2, and 3-year survival rates to be 37.2%, 31.0%, and 31.0%, respectively. The role of chemotherapy and radiation, be it in an adjuvant or palliative setting, remains poorly defined. In the present case, the AIM regimen was considered due to success in treating soft tissue sarcomas.^7^ Wada et al.^8^ reported on a patient who received 3 years of gemcitabine after curative resection and was still alive at the 5-year mark. Unfortunately, our patient’s tumor was unresectable due to its advanced state at presentation and chemotherapy could never be initiated due to an ongoing infection.

## Conclusion

Sarcomatoid carcinoma of the gallbladder is an exceedingly rare disease. Consequently, there is limited information on the disease process and no consensus regarding its treatment. For early-stage cases, surgical resection offers the best prognosis and is potentially curative, though many still develop local recurrence or metastasis. Unfortunately, most patients present with advanced disease and are often not surgical candidates. The role of chemotherapy and radiation in this population remains undefined and warrants further study. In the present case, the patient was not a surgical candidate due to his heavy tumor burden with locally advanced, invasive disease. Palliative chemotherapy was planned; however, our patient was never able to receive therapy due to complications from the underlying disease process.
